# Chitosans and Nanochitosans: Recent Advances in Skin Protection, Regeneration, and Repair

**DOI:** 10.3390/pharmaceutics14061307

**Published:** 2022-06-20

**Authors:** Patricia Garcia Ferreira, Vitor Francisco Ferreira, Fernando de Carvalho da Silva, Cyntia Silva Freitas, Patricia Ribeiro Pereira, Vania Margaret Flosi Paschoalin

**Affiliations:** 1 Programa de Pós-Graduação em Ciências Aplicadas a Produtos para a Saúde, Faculdade de Farmácia, Universidade Federal Fluminense, Niterói 24241-000, RJ, Brazil; patricia.pharma@yahoo.com.br (P.G.F.); vitorferreira@id.uff.br (V.F.F.); 2 Departamento de Tecnologia Farmacêutica, Faculdade de Farmácia, Universidade Federal Fluminense, Niterói 24241-000, RJ, Brazil; 3 Departamento de Química Orgânica, Instituto de Química, Universidade Federal Fluminense, Niterói 24020-141, RJ, Brazil; fcsilva@id.uff.br; 4 Departamento de Bioquímica, Instituto de Química, Universidade Federal do Rio de Janeiro, Av. Athos da Silveira Ramos 149, Sala 545, Cidade Universitária, Rio de Janeiro 21941-909, RJ, Brazil; freitas.cs@pos.iq.ufrj.br (C.S.F.); patriciarp@iq.ufrj.br (P.R.P.); 5 Programa de Pós-Graduação em Ciencia de Alimentos, Instituto de Química, Universidade Federal do Rio de Janeiro, Av. Athos da Silveira Ramos 149, Sala 545, Cidade Universitária, Rio de Janeiro 21941-909, RJ, Brazil; 6 Programa de Pós-Graduação em Química (PGQu), Instituto de Química, Universidade Federal do Rio de Janeiro, Av. Athos da Silveira Ramos 149, Sala 545, Cidade Universitária, Rio de Janeiro 21941-909, RJ, Brazil

**Keywords:** chitosan/nanochitosan scaffolds, composites, antimicrobial, anti-inflammatory, antiaging, antitumorigenic, wound-healing properties, drug delivery

## Abstract

Chitosan displays a dual function, acting as both an active ingredient and/or carrier for pharmaceutical bioactive molecules and metal ions. Its hydroxyl- and amino-reactive groups and acetylation degree can be used to adjust this biopolymer’s physicochemical and pharmacological properties in different forms, including scaffolds, nanoparticles, fibers, sponges, films, and hydrogels, among others. In terms of pharmacological purposes, chitosan association with different polymers and the immobilization or entrapment of bioactive agents are effective strategies to achieve desired biological responses. Chitosan biocompatibility, water entrapment within nanofibrils, antioxidant character, and antimicrobial and anti-inflammatory properties, whether enhanced by other active components or not, ensure skin moisturization, as well as protection against bacteria colonization and oxidative imbalance. Chitosan-based nanomaterials can maintain or reconstruct skin architecture through topical or systemic delivery of hydrophilic or hydrophobic pharmaceuticals at controlled rates to treat skin affections, such as acne, inflammatory manifestations, wounds, or even tumorigenesis, by coating chemotherapy drugs. Herein, chitosan obtention, physicochemical characteristics, chemical modifications, and interactions with bioactive agents are presented and discussed. Molecular mechanisms involved in chitosan skin protection and recovery are highlighted by overlapping the events orchestrated by the signaling molecules secreted by different cell types to reconstitute healthy skin tissue structures and components.

## 1. A Brief Overview of Biopolymers

Natural polymers exist in many forms present in wood, leaves, fruits, seeds, insects, algae, crustaceans, and animal skins and have been employed in many industrial sectors, such as clothing, energy production, construction, furniture, paper, wastewater treatment, and bioremediation for thousands of years. More recently, these polymers have also been used in sophisticated applications, such as in food and biodegradable food packaging, as well as the production of fine chemicals, biomedical products, and pharmaceutical formulations. Natural polymers have also been used for human tissue reconstruction to recover, maintain, or improve organ and tissue functions. Each polymer’s structure and chemical characteristics dictate the behavior of composites applied to living organisms [1,2,3,4].

Edible polysaccharides can provide cells with energy-maintaining cellular functions, and structural polysaccharides, such as cellulose and chitin, are enrolled in the structural maintenance of plant cell walls or as part of arthropod, mollusk, and insect exoskeletons [5,6,7].

Organic chemists have widely studied polysaccharides, as they are abundant, cheap, enantiomerically pure, and derived from renewable technology. Not surprisingly, they are regularly used as raw materials in the chemical industry to produce polymers and substances in fine chemicals. In terms of global industrial production, carbohydrates rank second, only behind vegetable oils. Estimates indicate that nature produces around 17 × 10^10^ tons of biomass every year, of which humans use only 3.5% as food and in chemical transformations [8,9]. The high global demand for polysaccharides has increased their prices, similarly to other synthetic substances used in industry, such as low-molecular-weight solvents.

**Figure 1:**
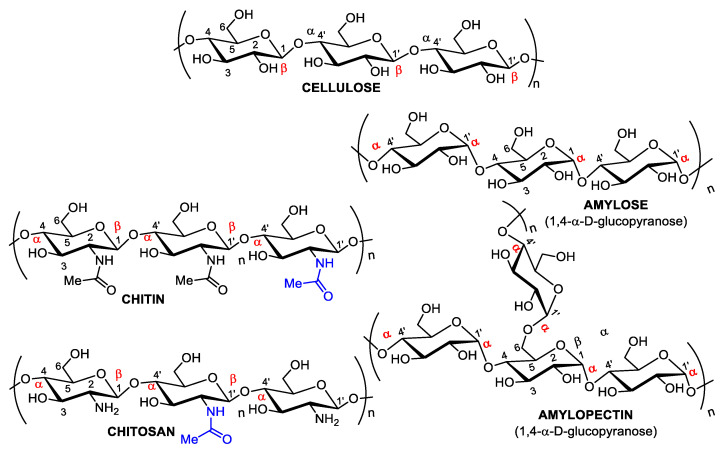
Major naturally abundant polysaccharides. Although cellulose, amylose, and amylopectin are D-glucose polymers, chitin and its deacetylated derivative, chitosan, are made up of D-glucosamine.

The main natural polymers are cellulose, starch, chitin, and chitosan (Figure 1); although alginates and fructans should also be considered, except for chitin and cellulose, all are edible and considered safe for human consumption.

First isolated by Braconnot [10], chitin and chitosan from mushroom cell walls, including edible species, are biocompatible, biodegradable, bioabsorbable, and non-toxic polymers. They are the two most abundant mucobiopolymers composed of randomly distributed *N*-acetyl-D-glucosamine (*N*-GlcNAc) monomers (Figure 1) [11,12,13]. These two polysaccharides are made up of a long chain of β-D-glucosamine joined together by the hydroxyls of anomeric carbons with a β configuration and four with an α configuration. These two polysaccharides are differentiated by their degree of N-acetylation of D-glucosamine, where chitin is poly-acetylated, and chitosan contains less than 50% (albeit variable) acetyl groups. The variation in the number of acetyl groups can be enzymatically modulated by chitin deacetylase, forming chitosan with different physicochemical properties [14]. Additionally, chitin treatment with concentrated protonic acids followed by heating produces high-yield D-glucosamine and its acetyl derivative, *N*-acetylglucosamine (NAG). Glucosamine, an amino sugar precursor for protein and lipid glycosylation, is used as a dietary supplement to ameliorate arthritis and pain, but both types promote healthy skin [15].

Although cellulose makes up most of the Earth’s biomass, chitin and chitosan have a twice as high replacement rate, making them economically attractive for the cosmetic and fine chemical industries. Widely available and cheap, these enantiomerically pure substances with known absolute configuration and derived from renewable technology are very attractive. Furthermore, the deacetylated polymeric form of chitosan exhibits increased solubility in protonated aqueous media. Finally, these compounds allow for the possibility of selective derivatization and hydroxyl manipulation by chemical or biochemical reactions and modification of the primary amino group on the chitosan backbone [16,17].

The scientific and technological developments with respect to chitin and its deacetylated polymer derivative, chitosan, obtained from biological sources and marine waste, comprising some of the most abundant in nature and commercially available D-glucosamine polymers, were updated. Chitosan chains can be manipulated and functionalized, generating molecules, chitosan composites, or nanochitosan composites with distinct physicochemical properties and biological activities. Novel pharmaceuticals formulated based on chitosan nanocomposites will be discussed, indicating their structural modifications, such as electrostatic interactions, chemical crosslinking, and metal ion coordination. In its different forms, chitosan can improve skin care product performance. However, chitosan composites, an eclectic class of biopolymers functionalized with different molecules, can be applied both for skin protection and skin health maintenance and to protect, treat, repair, and regenerate damaged skin following their inclusion in the formulation of various cosmeceutical, biomedical, and pharmaceutical products. In particular, chitosan nanocomposites may improve the pharmacokinetic behavior and therapeutic efficacy of traditional drugs while improving and widening their use and enhancing their inherent or acquired bioactivities to protect and regenerate or repair skin tissue. The molecular mechanisms involved in the benefic chitosan effects with respect to the recovery of injured skin tissue are highlighted, considering hemostasis, inflammation, migration, proliferation, and remodeling steps that support the reconstitution of healthy skin tissue structures and components, emphasizing the non-parenteral use of nanochitosan in skin moisturization against infection and oxidative imbalance and towards tissue regeneration and repair.

## 2. Chitin and Chitosan: Availability, Preparation, Physicochemical Characteristics, and Novel Pharmaceutical Uses

Chitin and chitosan originating from fungal and marine waste, such as crab or shrimp exoskeletons, and displaying low, medium, or high molecular weights and different deacetylation degrees are marketed by various manufacturers. The global chitin and chitosan market exhibited an annual growth rate (CAGR) of 15.4% between 2016 and 2020 and was valued at USD 42.29 billion in 2020. Estimates indicate that this figure will reach USD 69.297 billion in 2028, increasing at a CAGR of 5.07% from 2021 to 2028 (https://www.verifiedmarketresearch.com/product/chitin-market/, accessed on 17 November 2021). Companies conducting global market research estimate that this growth will be driven by novel biomedical applications, including pharmaceutical thickeners, protein entrapment, and the synthesis of biodegradable composites. However, additives in food and beverages, animal feed, agriculture, and water treatment may also contribute to the estimated increase. Many simple and inexpensive chemical methods for recovering these biopolymers from marine waste have been described in the literature, e.g., demineralization and deproteinization, which can extract chitin and chitosan from fungi and insects mollusk, shrimp, and crustacean shells. Yadav, et al. [18] reviewed the most important aspects of chitin and chitosan obtention methods from marine waste, as well as the preparation of nanostructured materials for commercial purposes. Chitin is obtained by chemical extraction processes, using acids to remove minerals and proteins. However, this method is not ecological and adversely affects chitin’s physical and chemical properties, although it is still the most applied method in large-scale industrial processes. Based on chitin’s similarity to cellulose, chitin nanofibers are produced according to protocols previously established for cellulose hydrolysis. Chitin can be hydrolyzed in acidic media and/or by enzymatic processes that cleave its crystalline structure [19], making subsequent chain deacetylation easy. Chitosans form when at least 50% of available deacetylated residues are obtained. Biodegradable and porous chitin and chitosan nanofibers under 100 nm in diameter display extremely high surface-to-volume ratios, presenting different morphological, optical, and mechanical characteristics. The most common method of obtaining chitin or chitosan nanofibers is through a disintegration process, employing either microwave irradiation or sonication [20,21,22].

Both chitin and chitosan are suitable for processing gels, membranes, nanofibers, spheres, microparticles, nanoparticles, scaffolds, and sponges [23,24,25]. Although nanostructured chitosans are less than 100 nm in size in at least one of their dimensions, they allow for increased interaction between components, making them more appropriate for human tissue recovery and repair engineering [26] as excipients in drug and gene delivery systems [25,27]. Nanochitosans exhibit three-dimensional architectural structures with open and interconnected pores that make them highly functional, resistant, and a suitable facilitator for drug encapsulation. In addition, they can also form inorganic composites that enhance their properties and ensure potential use in biomedicine tissue engineering, bone graft fabrication, and drug encapsulation, as indicated previously. Polymer/metal nanocomposites comprise a class of hybrid materials composed of an organic polymer matrix with dispersed metallic nanoparticles used in various applications, such as biosensors, cancer diagnosis/treatment, cell labeling, drug delivery, molecular imaging, and materials chemistry [28].

Chitosans are biocompatible with human tissues and present several pharmacological characteristics, including antimicrobial, hemostatic, anti-inflammatory, antioxidant, mucoadhesion, analgesic, adsorption-enhancing, antihypertensive, anticholesterolemic, anticancer, and antidiabetic properties. These intrinsic chitosan functionalities can be increased if sophisticated molecules are linked to chitosan scaffolds. In particular, chitosans and/or their counterparts can improve skin health and protection, aid in defense against carcinogenic radiation, oxidative stress, infections, and, eventually, skin cancer treatment. In the following sections, we will address the promotion of skin by chitosan repair by interfering with physiopathological conditions through gene expression control, cell adhesion, granulation tissue arrangement, and tissue re-epithelization.

## 3. Chemical Modifications on Chitin and Chitosan Surfaces: Developing Novel Pharmaceuticals

Chitosan exhibits several inherent pharmacological activities, such as antioxidant, antimicrobial, and antitumoral properties, that can be enhanced and broadened by chemical modifications or physicochemical interactions that contribute to healthy chitosan applications, particularly in benefiting and promoting skin tissue repair and regeneration. In their crystalline forms, the surface of chitosan and/or nanochitosans can be modified with different ligands comprising inorganic ions and hydrophobic or hydrophilic compounds. They can also be functionalized with very sophisticated molecules, such as antibodies, proteins, peptides, polysaccharides, and nucleic acids covalently linked to the primary molecule, or can act as carriers for these molecules through nanoencapsulation. Chitosan efficiency can be improved following nanoencapsulation, as nanochitosans display high surface/volume ratios [29].

Chemical modifications of chitin and chitosan functional groups can alter their physical and mechanical properties [30]. Many chemical reactions can insert functional groups on the surfaces of chitin and chitosan nanofibers to enhance, modify, or expand polymer functionality. Free amine groups are suitable for characteristic amine (NH) reactions that can be modified by acetylation/deacetylation, carboxyalkylation, phthaloylation, naphthaloylation, maleylation, chlorination, oxidation, and graft polymerization ranging from 5 to 8%. Secondary hydroxyl groups (OH) can also be chemically functionalized [31].

**Figure 2:**
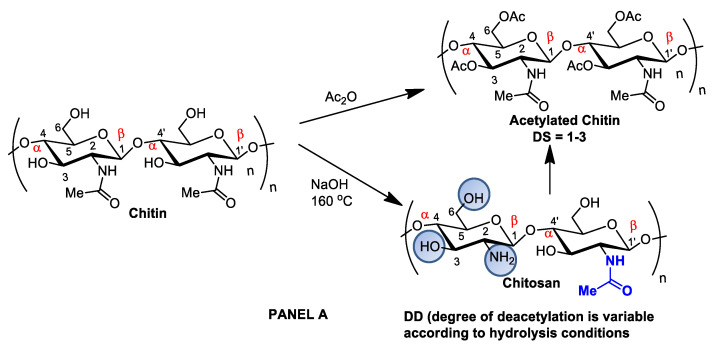

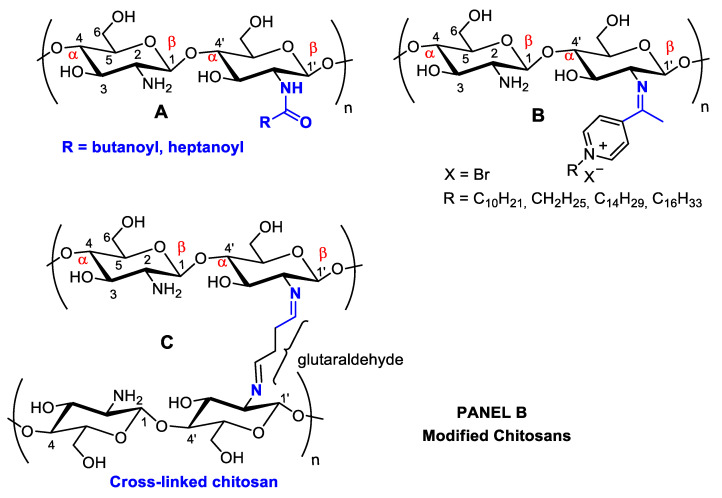
Panel (**A**): chitin and chitosan acetylation and deacetylation without alteration of the degree of polymerization. Chitin acetylation varies from 1–3 (DS = 1–3). Panel (**B**): chitosan structural modifications through amino group reactions with butanoyl or hepatanoyl (A), pyridine (B) or chitosan fibers crosslinking with glutaraldehyde (C).

Although chitin can be deacetylated by alkaline hydrolysis, other sustainable and ecofriendly methods have been developed to generate chitosan from crustaceous solid residues. The hydrolysis of the *N*-acetamido groups in chitin *N*-acetyl-D-glucosamine residues can also be performed by employing recombinant yeast chitin deacetylase (E.C.3.5.1.41), which converts chitin into chitosan in a controlled manner with no harsh secondary residues [19,32]. Recently, chitosan nanofibers were obtained by treating chitin crustaceans with the crude extract of Annona muricata, an edible plant originally from the Antilles [33]. Alternatively, chitin can be acetylated by introducing additional acetyl groups without altering the degree of polymerization, generating biopolymers for different applications. Chitin acetylation is the simplest functionalization to perform, but complete deacetylation is rarely achieved, and some remaining compounds retain some acetamide groups (Figure 2A).

The introduction of certain functional groups in the two chitin nanofiber hydroxyl groups can significantly improve their physicochemical properties and expand their use in new applications [34]. Polyacetylation and carboxymethyl acetylation, replacing hydrogens in the amino and hydroxyl groups, confer chitosan hydrophobicity and consequent waterproofing properties, which are very useful for obtaining non-toxic and biodegradable bandage coatings for minor cuts, scrapes, blisters, and burns, associating inherent chitosan healing properties to waterproof protection (Figure 2A—Panel A) [35,36].

However, chitin and chitosan acetylation degree, as well as other physicochemical characteristics, such as molecular mass and moisture content, are associated with fungal and bacteria growth inactivation. Low-molecular-weight chitosan oligomers pass through the cell membrane and alter cell permeability [37]. Nanochitosans have been shown to disassemble bacteria cell membranes and inhibit metabolic and biosynthetic pathways [38].

**Figure 3:**
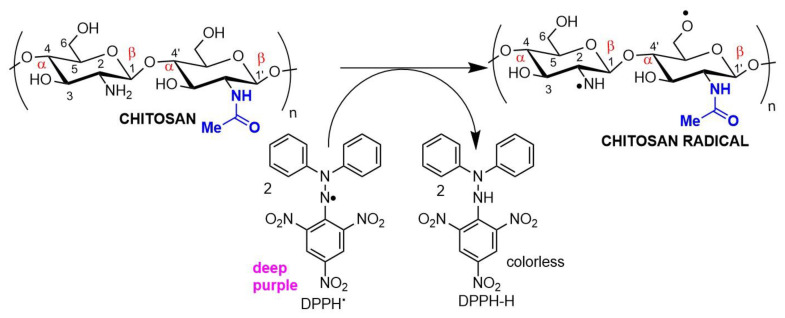
Chitosan radical scavenger activity demonstrated in an antioxidant assay using DPPH (2,2-diphenyl-1-picrylhydrazyl) as the electron acceptor.

Antioxidant chitosan properties are ascribed to free amino groups, amino acetyl groups, and hydroxyl groups found in polymer molecules, all of which can react with different reactive oxygen (ROS) and nitrogen (RNS) species (Figure 3). Many studies indicate that chitosan polymers display antioxidant activities. However, the molecular mechanisms of chitosan radical scavenger abilities are still not well understood from a chemical point of view [39,40]. The antioxidant activity of chitosan increases with the degree of acetylation and with decreasing biopolymer molecular weight. It is important to note that shorter chains form fewer intramolecular hydrogen bonds between hydroxyls, resulting in more activated hydroxyl and amino groups, contributing to radical scavenging activity. Chitosan antioxidant activities can be identified by employing a very common antioxidant assay involving 1,1-diphenyl-2-picrylhydrazyl (DPPH) radicals. The DPPH radical exhibits a strong absorption band centered at about 520 nm and becomes colorless or pale yellow when neutralized (Figure 3). The ability to scavenge the DPPH radical by chitosan ranges between 30 and 60% [41].

The antioxidant/antimicrobial effects of chitosan and its derivatives can be explored in drug and cosmetic formulations due to their biocompatibility, non-toxic character, and effective human metabolization, enhancing the safety and shelf life of both pharmaceuticals and food products [42].

**Figure 4:**
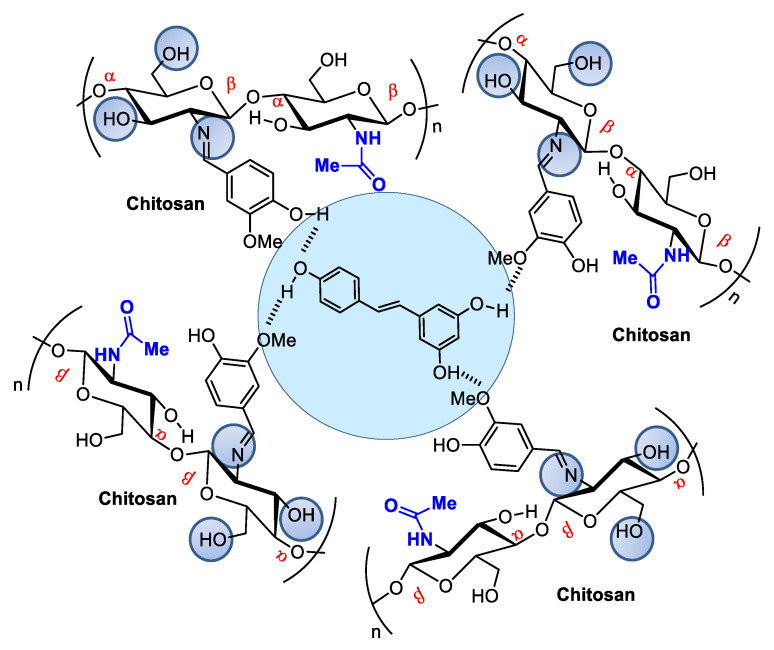
Resveratrol encapsulated in chitosan microspheres crosslinked with vanillin can be administered to modulate metabolic dysfunctions.

When associated with resveratrol (3,5,4′-trihydroxystilbene), a natural phytoalexin, chitosan synthesized in cis and trans isomers of grapevines in response to pathogen infection or physical injury can be administered either topically or systemically (Figure 4). Resveratrol owes its antioxidant activity to its phenolic hydroxyl groups and can be used to achieve oxidative skin cell homeostasis following topic use or aid in the fight against chronic and degenerative human physiopathological conditions by promoting organism oxidative balance when administered systemically as a nutraceutical or pharmaceutical [43].

Resveratrol from red wine has been considered promising in preventing human cardiovascular diseases [44] and as an antitumorigenic [45,46] and antiviral [47] compound, among other applications. It also modulates lipid metabolism and inhibits low-density lipoprotein oxidation and platelet aggregation. The potential of this natural product has led to hundreds of studies concerning the search for pharmaceutical strategies for its stabilization and use in food and cosmeceutical supplements. Regarding controlled release, many studies describe resveratrol incorporation into several natural and modified chitosan types in film, nanoparticle, or microsphere form [48,49,50]. For example, Frémont [51] incorporated resveratrol into chitosan microspheres crosslinked with vanillin. The microspheres presented a smooth surface composed of small but irregularly sized particles ranging from 53 to 311 μm. Network crosslinking between chitosan and vanillin occurred via the Schiff base reaction, and resveratrol encapsulation efficiency in the chitosan microsphere reached an efficiency rate of up to 93.68%.

Sodium tripolyphosphate (TPP) salts can also be used to encapsulate resveratrol in microspheres at concentrations that considerably alter its bioavailability, consequently increasing its effectiveness [52].

Chitosans also exhibit well-documented antimicrobial activities against Gram-positive and Gram-negative bacteria and fungi [38,53]. However, due to its low solubility, antimicrobial activity is limited to acidic media, although antimicrobial activity in neutral media can be improved by transforming amino groups into quaternary ammonium salts (NR4 + X^−^) [54]. Structural chitosan modifications obtained by introducing groups covalently linked to amines or hydroxyls can increase antimicrobial and antioxidant activities compared to native chitosan [55].

**Figure 5:**
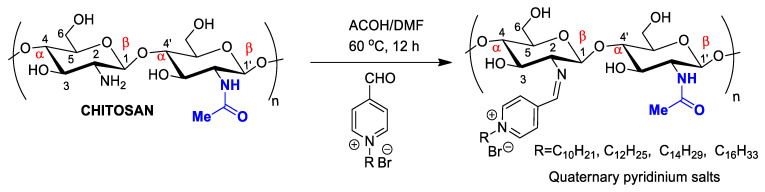
Chitosan nanoparticles functionalized by long-chain pyridinium salts to increase antimicrobial effectiveness.

Chitosan antimicrobial activity can be customized and enhanced by covalently binding to antibiotics or antifungal agents to treat nosocomial infections. The introduction of radicals containing quaternary ammonium/pyridinium salts used as commercial antimicrobial agents (e.g., dimethyl dodecyl ammonium chloride or bromide and cetylpyridinium chloride) can improve antimicrobial activity (Figure 5). For example, chitosan nanoparticles modified through the formation of a Schiff base in the amino group, forming long-chain pyridinium radicals, exhibited increased antibacterial activity against the Gram-positive species *Staphylococcus aureus* and *Bacillus cereus*, as evaluated by MIC (minimum inhibitory concentration) and MIB (minimum bactericidal concentration), as well as mild antioxidant effects (Figure 3) [56].

Metallic nanoparticles containing Ag, Ni, Cu, Pt, Co, Pd, or Au can exhibit unusual physical and chemical characteristics distinct from their bulk counterparts or individual metals [57], such as antimycotic, antiparasitic, antineoplastic, insecticidal, and antibiotic properties [58]. For example, Ag-chitosan nanoparticles are promising pharmaceuticals to treat fungal infections caused by *Candida* spp., with fungal growth inactivation due to cytoplasmic degeneration and membrane and cell wall rupture. Ag-nanochitosans in combination with fluconazole and amphotericin B demonstrated an additive effect. Low nanocomposite cytotoxicity against mammalian cells has been reported in *Galleria mellonella* larvae, suggesting potential in vivo use. When tested as a topical treatment against murine cutaneous candidiasis, nanocomposites were found to reduce fungal skin loads in a dose-response behavior and favored tissue repair [59].

**Figure 6:**
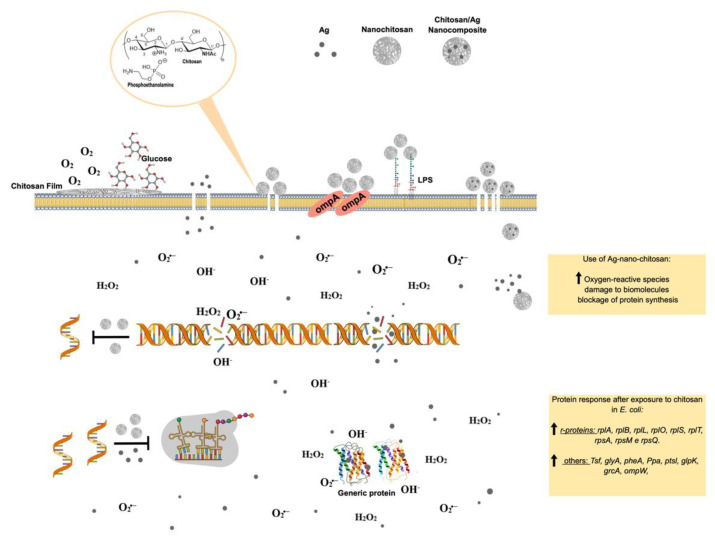
Molecular effects of chitosan nanoparticles on *E. coli* growth inactivation. Chitosan seems to interact with phospholipids, particularly phosphatidylethanolamine (PE), the most abundant phospholipid in *E. coli*, triggering the impermeabilization or disassembly of its outer membrane. The impermebialization of bacteria membrane by the nano-chitosan films inhibit glucose and O_2_ uptake, causing bacteria death. After exposure to nano-chitosan particles, *E. coli* experiences a differential expression of proteins involved in protein biosynthesis machinery and amino acid and purine nucleotide supply, as already demonstrated [38] and shown in the yellow rectangle at the lower right corner. When *E. coli* is exposed to Ag-nano-chitosans, an increase in reactive oxygen species is observed, followed by consequent damage to cellular structures, including proteins and nucleic acids. Chitosan and Ag nanoparticles, as well as cellular structures, are not represented in their real scale. This figure was drawn using the infographic maker Mind the Graph, available at https://mindthegraph.com.

Molecular chitosan antimicrobial enrollment mechanisms are not yet fully understood. The most accepted mechanism involves the combination of chitosan cationic amine groups and anionic phospholipids on bacterial surfaces, promoting membrane disassembly and cell disintegration (Figure 6) [60]. It has also been proposed that chitosan and its low-molecular-weight derivatives, penetrate the bacterial membrane, thus impairing selective protein biosynthesis [38,61]. Another possibility is that chitosan, employing its exceptional ability to bind metals, interacts with divalent ions in bacterial cell walls to prevent cells from performing their normal functions [62]. Chitosan can also act as an occluding compound, docking to the outer bacteria membrane and preventing nutrient cell uptake and oxygen diffusion (Figure 6) [63]. Many studies have focused on optimizing the antibacterial properties of chitosan through various modifications [56,64]. For example, Liu, et al. [65] reported that surface grafting of gentamicin molecules improved antibacterial chitosan properties [65]. In another study, chitosan films incorporated with Ag nanoparticles exhibited enhanced antimicrobial properties (Figure 6) [66]. Chitosan’s wound-healing properties are enhanced in Ag-chitosan. Chitosan/glycosaminoglycan scaffolds loaded with Ag nanoparticles can also promote wound repair by stimulating fibroblast proliferation [67] and modulating the matrix production of metalloproteinases, preventing degradation of both fiber junctions and peptide growth factors [68]. Other studies report that nanofibers with Ag nanoparticles promote wound healing by activating the TGF β1/Smad signaling pathway [69]. Furthermore, the addition of Ag nanoparticles can effectively and conclusively inhibit an inflammatory response by regulating inflammatory mediators, and Ag-chitosan has been proven to induce a superior anti-inflammatory inhibitory effect than any monomer, significantly reducing interleukin-1 levels [70].

Chitin derivatives exhibiting controlled molecular weight and/or deacetylation degree variations have been reported to display antitumorigenic activity against several cancer cell lines, including human myeloid leukemia HL-60 cells, human melanoma cells (cell lines A375, SKMEL28, and RPMI7951), HeLa cells, human hepatoma, and human monocyte leukemia cells [71,72,73,74,75].

Schneible, et al. [76] studied drug delivery systems employing chitosan hydrogels for combined chemotherapy using doxorubicin and gemcitabine for the synergistic treatment of advanced solid tumors (i.e., breast cancer). Butanoyl and heptanoyl-hydrophobic chains were chosen for chitosan modification to target drug release kinetics to control the hydrogel network structure and, therefore, drug migration (Figure 2A). Specifically, these modifications allow for modulation of the molar ratio and relative drug release kinetics, which has been validated experimentally, allowing for the prediction of behaviors for specific formulations offering synergistic effects. The achieved results demonstrate the potential of this approach to accelerate the use of drug-laden hydrogels for cancer therapy, including chitosan-doxorubicin, chitosan-gentamicin, chitosan-butanoyl, heptanoyl hydrophobic chains, dimethyl dodecyl ammonium chloride, or bromide and cetylpyridinium chloride (Figure 5). Many substituents can be inserted into hydroxyl groups, depending on the intended therapeutic effect. For example, chitosan can be crosslinked using glutaraldehyde (Figure 2B) [77]. The more rigid structure of this polymer crosslinked with a dialdehyde linking the NH_2_ groups can be used for many different applications compared to isolated chitosan fibers, mainly concerning polymer strength. This material can be coated with other polymers, forming surface films bonded by hydrogen bonds. With the aim of increasing the resistance of crosslinked polymers, Sun, et al. [78] anchored poly(ethylene oxide) presenting different molecular weights when preparing various membrane materials, and the surface of this dense-structure membrane became less porous, decreasing the amount of water absorption and pore size and increasing thermal stability and tensile strength.

## 4. Chitosan Can Be Combined with Other Polymers to Build Broader Drug Carriers

To broaden its application but maintain its non-toxic character, chitosan can be combined with other natural biopolymers, such as dextran sulfate, carrageenan, Arabic gum, glucomannan, cellulose, pectin proteins, and even DNA, among others [79], whereas alginate, agarose, and collagen have been successfully combined with chitosan/nanochitosan.

### 4.1. Alginate and Chitosan Composites

**Figure 7:**
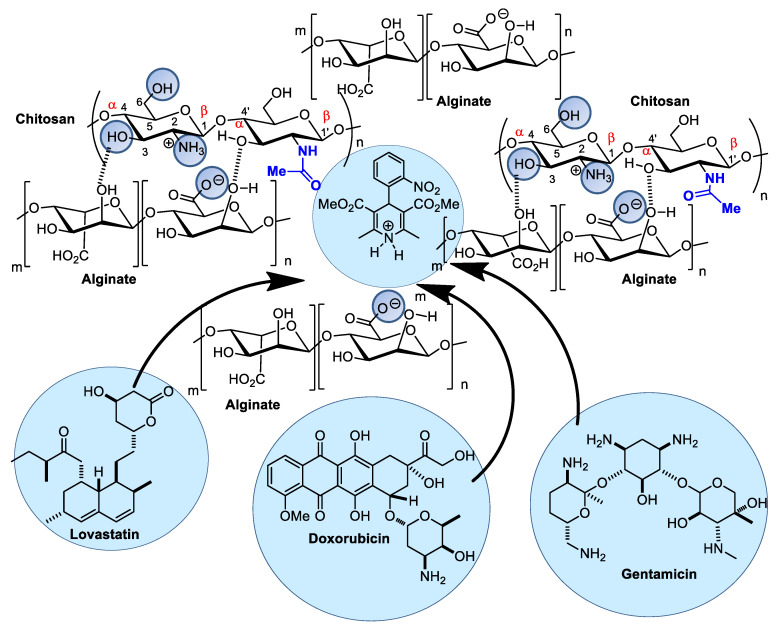
Alginate/chitosan nanocomposites as drug carriers can load several pharmaceuticals, such as lovastatin, doxorubicin, and gentamicin.

Alginic acid and alginate are polysaccharides of variable molecular masses composed of two monomers in a 1-4 linkage between α-L-guluronic acid and β-D-mannuronic acid found in the cellular structures of certain bacteria and brown seaweeds. Both monomers are uronic acid derivatives. They can form salts with different metallic cations and colloidal or gelatinous solutions in aqueous media at an adequate pH, making these biopolymers suitable for use in cosmetic, cosmeceutical, and pharmaceutical formulations. In pharmaceutics, alginate nanoparticles can be combined with other polymers, forming supramolecular interactions employed as drug delivery systems. These polymer associations display several advantages in drug encapsulation. Alginate/chitosan nanocapsules can accommodate hydrophobic molecules presenting different molecular weights and structural scaffolds [80,81,82]. Some examples are highlighted in Figure 7, which illustrates the interactions of several drugs with chitosan/alginate nanocapsules. A significant example related to wound healing comprises a nanofibrous alginate/chitosan dressing containing gentamicin. This fibrous material displayed high antibacterial performance superior to that of gentamicin alone associated with improved wound adhesion, cell proliferation, and wound regeneration when tested on Balb/C mouse skin [65,83]. Another hydrophobic compound, the antihypertensive nifedipine, was efficiently nanoencapsulated in sodium alginate:CaCl_2_:chitosan with a mass ratio of 30:6.7:3.2 (*w*/*w*/*w*). These biopolymer associations were shown to be efficient in rapid in vitro release, followed, as expected, by a controlled release. More recently, alginate/chitosan nanoparticles were used as a carrier for lovastatin and analyzed by Fourier transform infrared spectroscopy (FT-IR), scanning electron microscopy (SEM), laser scattering, and differential scanning calorimetry (DSC) [84]. Spectroscopic data indicate that hydrogen bonds and dipolar–dipolar interactions between lovastatin and alginate/chitosan nanoparticles occur inside these spherical nanocapsules. The melting temperature of alginate/chitosan/lovastatin nanoparticles was higher than that of chitosan and lower than that of alginate separately. The drug release process also involved a rapid release stage followed by a slow release stage. Alginate and chitosan can also substitute nanoliposomes to carry antineoplastic drugs such as doxorubicin, which binds to DNA-associated enzymes by intercalation in DNA base pairs. Alginate-chitosan-doxorubicin nanocapsules exhibited a superior cytotoxic effect on multidrug-resistant lymphoma cells (L5178 MDR1) but reduced cardiotoxic effects on H9c2 cardioblasts in culture [84].

### 4.2. Chitosan and Agarose Composites

**Figure 8:**
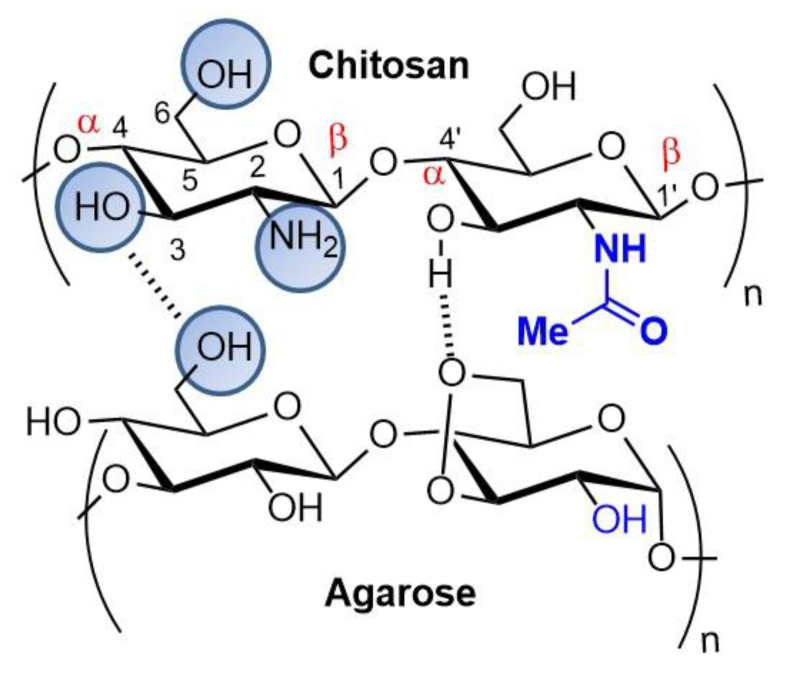
Chitosan and agarose scaffolds.

Functional chitosan and agarose composites, as well as linear and stable chains of polysaccharides, extracted from marine red algae composed of alternating units of β-D-galactose and 3,6-anhydrous-L-galactose, can be built, and the ratio of each polymer confers different composite properties according to the desired application. The polysaccharides are similar in their three-dimensional structures and are capable of hydrogen bonding (Figure 8) [85,86]. Due to its predominantly hydrophilic and macroporous structure, agarose can form gels similar to some human tissues, and chitosan-agarose composites exhibit excellent mechanical properties coupled with superior antibacterial activities relative to those of pure chitosan structures [87].

### 4.3. Chitosan and Collagen Composites

Collagens, such as chitosan, are also abundant biopolymers of considerable importance in organismal structures. Although they do not exist together as mixtures in nature, cellular structures obtained from chitosan or collagen are similar in composition and mechanical behavior to many human tissues. Chitosan and collagen exhibit structural properties capable of interacting with each other through the interaction of hydrogen bonds, producing artificial mixtures with special rheological and mechanical features that are suitable for use as support materials for extracellular matrix regeneration in mammals. These supramolecular chitosan and collagen structures can be used in tissue recomposition, including skin recomposition [88,89]. Recently, chitosan and collagen composites printed by a three-dimensional (3D) printer were used for tissue regeneration. Neither of the biopolymers alone are suitable for use as a material for 3D printing, as they need to form non-brittle layers and can overlap; however, chitosan-collagen composites display rheological viscosity and gelation temperature characteristics suitable for 3D printing. The authors concluded that adjusting chitosan ratios decreased printed layer swelling rates and increased fiber tensile strength, making these composites suitable for the manufacture of different kinds of biological tissues, depending on the desired application [90].

Chitosan and collagen composites can also be modified with the addition of a third component comprising organic or metallic compounds, such as silver, resulting in novel properties for these composites [91]. Tannic acid, a non-toxic, naturally occurring compound, can be used for the safe crosslinking of both polymers [92].

Additionally, Lin et al. [93] synthesized and characterized spongy materials composed of collagen/hyaluronic acids/chitosan for potential biomedical applications.

## 5. Chitsosan and Nanochitosan Skin-Targeted Products: Novel Cosmetics, Cosmeceuticals, and Pharmaceuticals

In cosmetic science, both chitin and chitosan have been investigated as excipients and active biological agents, mainly due to certain specific properties, such as lack of toxicity, biocompatibility, and biodegradability. Chitin nanofibrils, as the purest crystal form of chitin, have positive surface charges and can electrostatically interact with electronegative bioactive compounds, generating innovative antiaging cosmetics and novel formulations to treat sensitive skin [94]. Additionally, these positive charges interact electrostatically with the stratum corneum, altering the thin lamellar layers and allowing for better diffusion through the skin of trapped active particles. The combination of electropositive chitin nanofibrils and electronegative polymers, such as nanolignin and hyaluronic acid, gives rise to nano/microcapsules capable of trapping hydrophilic and lipophilic molecules, such as vitamins and microelements, as well as anti-inflammatory compounds, antioxidants, antiaging substances, immunomodulatory agents, and enzymes [95]. This biopolymer combination results in cosmetics and cosmeceuticals that can be used for several purposes, including ultraviolet (UV) protection or hair care, but mainly to hydrate, rejuvenate, or lighten the skin and repair skin in the case of injuries [94,96].

Nanomaterial-based cosmetics, cosmeceuticals, and pharmaceuticals designed for skin exhibit many advantages compared to microscale cosmetics, as they are long-lasting and display relatively higher stability. The high surface area of nanomaterials allows for more efficient ingredient transport across the skin, providing new shade and color elements, which are very important in lipsticks and nail polish, as well as transparency, a desirable feature for sunscreens [97]. Biopolymers formed by chitosan and nanolignin can be used to develop hair tissue products to protect the hair and scalp from the environment, as well as specific aggressive cosmetic treatments, possibly also inducing self-repairing structural activity. When functionalized, these intelligent and innovative fabrics are natural carriers of bioactive compounds, eliminating other preservatives and emulsifiers, as well as other types of aggressive chemicals [98].

Chitin nanofibers can form an amalgam with active pharmaceutical and cosmeceutical complexes, improving their bioactivities [99,100,101]. However, their natural characteristics make these polysaccharides excellent candidates for cosmetic formulations, for example, antimicrobial (bacteria and fungi) and antioxidant activities, due to their scavenging capacity against different oxygen radicals species, such as alkyl, superoxide, hydroxyl, and DPPH (2,2-diphenyl-1-picrylhydrazyl), as well as mucoadhesive properties and penetration, associated with the opening and destruction of tight epithelial junctions [102,103]. Their antimicrobial activity, low antigenicity, and ability to induce wound healing can enhance the quality and efficiency of products molecularly designed for skin use [104]. Additionally, it is very common for cosmeceuticals to provoke skin hypersensitivity, making it paramount to develop materials with no proinflammatory activity. The inherent antimicrobial and anti-inflammatory activities of chitosan and its derivatives can replace other preservatives in cosmetics, reducing formulation allergens and avoiding inflammatory manifestations (Table 1) [105].

In addition, multipurpose nanochitosans can be obtained for skin treatment, as these compounds can be formulated as gels, fibers, or porous matrices that can be used for skin and mucous membrane hydration for antiaging or antiacne cosmetic therapy (Table 1) [94].

Regarding skin hydration, nanochitosans in emulsions can generate a hygroscopic molecular film that delays water evaporation and contributes to skin hydration [106,107].

Chitin-derived nanofibrils and nanocrystals were found to improve granular epithelial layers and increase granular density in a three-dimensional skin culture model and Franz cells, making them excellent candidates for nanocosmetics and personal care products [108]. The protective skin effect seems to be due to reduced TGF-β levels [30].

When nanochitosans encounter the stratum corneum, they are hydrolyzed by skin enzymes into smaller units, disaccharides, and tetrasaccharides, which can undergo the repolymerization process once they reach the inner epidermis and dermis layers. On the other hand, complete hydrolysis of nanochitosans results in the release of glucosamine, and its acetyl derivative *N*-acetylglucosamine molecules are involved in diverse cellular metabolic pathways and are critical for health of the body, particularly the skin. In turn, glucosamine can be further catabolized to supply the systemic cellular metabolism with its precursors, glucose and glutamic acid, whereas locally, in the extracellular dermal matrix, it can limit water loss by retaining water molecules. Glycosaminoglycans synthesized from glucosamine and *N*-acetylglucosamine are involved in dermis hydration maintenance; on the other hand, they represent the monosaccharide unit responsible for the structural stability of the extracellular dermal matrix. *N*-acetylglucosamine can reduce skin hyperpigmentation and therefore reduce the appearance of blemishes by inhibiting melanin production, which is effective in reducing wrinkles and acne, as well as increasing skin exfoliation and skin hydration [94]. Additionally, *N*-acetylglucosamine controls collagen synthesis during wound healing, promoting granulation tissue and skin lesion repair [96,109].

Concerning sunscreen development, nanochitosan in multifunctional emulsions can act as an active carriers and/or potentiator of antioxidant and immunomodulatory compounds, such as lutein, melatonin, and ectoin, to enhance the photoprotective effect against UV-induced damage. Nanoparticles combined with urocanic acid, a natural protective agent against UVB radiation, have been proven more effective than conventional products. Additionally, the protective effect of NCs against UV radiation can be attributed to their ability to reduce TGF-β (transforming growth factor β) production, a multifunctional cytokine responsible for regulating the inflammatory response [108].

Chitosan-alginate nanoparticles are very promising for the treatment of acne affections, as they display in vitro antimicrobial activity against *Propionibacterium acnes*, the bacteria associated with acne pathogenesis. These nanocopolymers inactivate *P. acnes* growth by inducing bacterial cell membrane disruption (Figure 5), promoting an anti-inflammatory reduction in cytokine production in human monocytes and keratinocytes. Furthermore, benzoyl peroxide (BP), one of the most common topical prescription medications for acne, has been effectively encapsulated in chitosan-alginate nanoparticles, displaying superior antimicrobial activity against *P. acnes* compared to BP alone, demonstrating less toxicity to eukaryotic cells. Together, these data suggest the potential utility of topical delivery of chitosan-alginate nanoencapsulated drug therapy to treat infectious dermatologic conditions accompanied by inflammatory manifestations [110].

When nanochitosans are complexed with hyaluronic acid, they acquire specific biological abilities, such as the ability to promote fibroblast proliferation and cytokine modulation. Cosmetic emulsions based on this natural polymer promote skin amelioration through anti-inflammatory and elasticity effectiveness and decrease skin hyperpigmentation, thus improving antiaging results [94,111]. Chitosan and hyaluronic acid can form nanolamellae or nanoparticles, which exhibit high surface ratios, enabling them to interact with enzymes, platelets, and other cellular components found in living skin tissue, resulting in the acceleration of the cutaneous granulation phenomena accompanied by angiogenesis and collagen fiber deposition, with consequent repair of dermal–epidermal lesions. [112].

In a recent study, a protective effect of chitin nanofibers on epithelial cells was demonstrated. Although this molecular mechanism is not yet fully understood, the effect has been attributed to the ability of chitin nanofibers to prevent moisture evaporation [88]. Additionally, applying chitin nanofibers to skin led to lower production of TGF-β compared to the control group. The cytokine TGF-β is a suppressive factor that regulates cellular processes related to wound and skin inflammation. These results suggest that chitin nanofibers may exhibit a natural antibacterial protective effect, maintaining skin hydration but inducing less inflammation. Wound size reduction, histological examination, and Smad3 expression, as well as the quantities of transforming growth factor-β1, tumor necrosis factor-α, and interleukin-8 protein, were measured to evaluate potential healing effects. The results demonstrate that *N*-carboxymethyl chitosan efficiently accelerated wound healing via activation of the transforming growth factor-β1/Smad3 signaling pathway [113].

Nanochitosans are also used to produce biodegradable face masks, as they are non-toxic and compatible with human keratinocytes. It is important to emphasize that chitin exhibits a differentiated ability to modulate the inflammatory cascade, depending on –NH_2_ and –OH groups and molecular weight. Chitin fragments smaller than 40 µm induce cytokine production, inhibiting tissue inflammation. Medium-sized chitin (40–70 µm) induces a proinflammatory response, whereas larger sizes have been reported as inert [114,115].

**Table 1:** Studies on chitosan formulations for skin protection and cosmeceutical applications.

Chitosan or Chitosan Composite	Type of Assay	Biological Effect	Experimental Conditions	Ref.
Chitin nanofibril-hyaluronan block copolymeric nanoparticles (CN-HA)	In vitro In vivo (healthy women)	Antiaging activity	Eye cream applied twice a day and serum: 2/3 drops twice a day, three times a week for 60 days	[94]
Chitin nanofibers and nanocrystals	In vitro	Skin protective effects	Nine tested conditions using nanofibers and nanocrystals at 4, 12, and 24 h post-application	[108]
Chitosan-alginate nanoparticles	In vitro	Antimicrobial and anti-inflammatory activity	1%, 0.5%, 0.2%, and 0.1% of chitosan-alginate nanoparticle/4 h	[110]
Chitosan nanoparticles	In vitro	Antimicrobial activity	0.5 and 1 mg/mL chitosan derivatives/24 h	[61]
Silver-nanoparticle-incorporated chitosan-based membranes	In vitro In vivo (rat)	Antibacterial efficacy and wound-healing ability	12 mg and 60 mg silver nitrate/chitosan-based membranes for 7 and 28 days	[116]
Chitosan nanoparticle-containing dexamethasone sodium phosphate	In vitro	Anti-inflammatory activity	4.19, 10.65, and 43.06% of dexamethasone/5 mg chitosan nanoparticles for 35 days	[100]
Phosphatidylcholine hyaluronic acid chitin–nanofibrils complex	In vivo (volunteer patients)	Antiaging activity	Single injection (1 mL solution with 10 µg/mL block-polymer) every 7 days for 10 weeks	[111]
Chitin nanofibril-hyaluronan nanoparticles (CN-HA)	In vitro and in vivo studies (women)	Antioxidant and anti-inflammatory activities	2 mg/mL CN-HA nanoparticles for 60 days	[103]
Carvacrol and eugenol chitosan nanoparticles	In vitro	Antioxidant activity	0.125 mg/mL to 1 mg/mL	[117]
Chitosan nanoparticles	In vitro	Drug delivery applications	30% *w*/*w* (protein based on chitosan)/12 mL of phosphate buffer	[101]
Chitosan nanofibers	In vivo (mice)	Antileishmanial wound	20 wt% nanofibers as wound dressings, daily for 30 days	[104]
Chitin nanofibrils	In vivo (rat)	Wound healing	2 g/L chitin nanofibril for 15 days	[109]
Chitin nanofibril-hyaluronan block copolymeric nanoparticles (CN-HA)	In vitro In vivo (healthy women)	Antiaging activity	Eye cream: applied two times a day; serum: 2/3 drops two times a day, three times a week (60 days)	[94]
Chitin nanofibers and nanocrystals	In vitro	Skin protective effects	Nine tested conditions using nanofibers and nanocrystals at 4, 12, and 24 h post-application	[108]
Chitosan-alginate nanoparticles	In vitro	Antimicrobial and anti-inflammatory activities	1%, 0.5%, 0.2%, and 0.1% of chitosan-alginate nanoparticles/4 h	[110]
Chitosan nanoparticles	In vitro	Antimicrobial activity	0.5 and 1 mg/mL chitosan derivatives/24 h	[61]
PVA/Chitosan hydrogel dressing loaded with PHMB	1: In vitro 2: In vivo (dog)	1: Growth inactivation of *S. aureus* and *S. epidermidis* 2: Faster wound recovery	5% PVA/chitosan (1:1) + PHMD 8.12 µg/mg dry sample Daily topical application for 21 days	[118]
Chitosan dressing–loaded iturin-AgNPs (CS-AgNPs)	In vivo (mice)	Faster wound healing and reduced *E. coli* colonies	Wounds covered with 0.02 g/mL CS and iturin-AgNPs 10 μg/mL	[119]
2 chitosan-dialdehyde cellulose (2CS-DC) composite foam sponge	In vivo (rabbit)	Reduced hemostasis time by 79.5% or 47.7%	Amputated tail covered with 2:1 CS-DAC 0.02 g Femoral vein excision covered with 2:1 CS-DAC 1.0 g	[120]
Oligo-chitosan (O-C) scaffold	Ex vivo (blood of (vWD) patients)	Increased release of PDGF and TGF-β1 by 29.8% and 23%, respectively; platelet activation, adhesion, and aggregation promotion	O-C 75–95% DDA applied to the blood	[121]
Chitosan/titanium dioxide (CS/TiO_2_) composite membrane	In vitro	Fibroblast proliferation; increased cytokine expression; *S. aureus* growth inactivation	CS/TiO_2_ membrane incorporated with 025% TiO_2_	[122]
Chitosan-PVA soft membranes plasticized with glycerol	In vivo (rabbit) In vitro	Burn wound healing in second-degree burns; inhibition of *P. aeruginosa*, *E. coli*, and *B. subtillis*	Chitosan 80%, PVA 20%, and glycerol 2%	[123]
Chitosan mesh membrane wound dressing	Clinical trial (skin donor patients)	Faster wound healing with no scar formation on the 10th day	1% chitosan mesh membrane covering the wound for two months	[124]
Chitosan sheet wound dressing	Clinical trial (skin donor patients)	Faster wound healing on the 11th day	No posology informed; wounds treated with chitosan sheets for six months	[125]
Heparin-chitosan membrane wound dressing	Clinical trial (skin donor patients)	Wound-healing acceleration on the 12th day	Heparin 7% in 1% chitosan membrane covering the wound with 15-day follow up	[126]
Chitosan-capped silver nanoparticles	In vivo (rat)	Burn wound healing	Ch/AgNPs to 50 mg/wound of the 1% silver sulfadiazine for 28 days	[70]
Silver chitosan nanocomposites	In vitro In vivo (mice)	Antifungal	Nanocomposites: 0.06 to 16 μg/mL 3 to 5 μg/mL of nanocomposites for 4 days	[59]
Chitosan-gentamicin (CS-GS) film	In vitro	Antibacterial—*S. aureus* and *E. coli*	CS-GS films immersed in PBS for 1, 3, 5, and 7 days were covered with 0.5 mL Log-phase bacteria suspension for 24 h	[65]
Chitosan/glycosaminoglycan scaffolds-Ag Nanoparticles	In vitro	Antimicrobial—*S. aureus* and *E. coli* Human fibroblast proliferation	Scaffold portion with Ag 385 μg/mL added to bacteria suspension for 24 h; 0.36 cm^2^ scaffold portions added to fibroblast culture for 3 and 6 days	[67]
Silver nanoparticles/chitosan oligosaccharide/PVA nanofibers	In vivo (rat)	Wound healing	5% nanofibers for 18 days	[69]

PVA—polyvinyl alcohol; PHMB—polyhexanide; Ch—chitosan; DDA—degree of deacetylation; vWD—Von Willebrand disease.

## 6. Molecular and Cellular Skin Repair Mechanisms of Chitosan-Based Wound Dressings

Biocompatibility, biodegradability, non-toxicity, and other physicochemical features make chitosan and its derivatives suitable for repairing injured skin due to their overall effects on skin architecture, as described previously [127,128].

**Figure 9:**
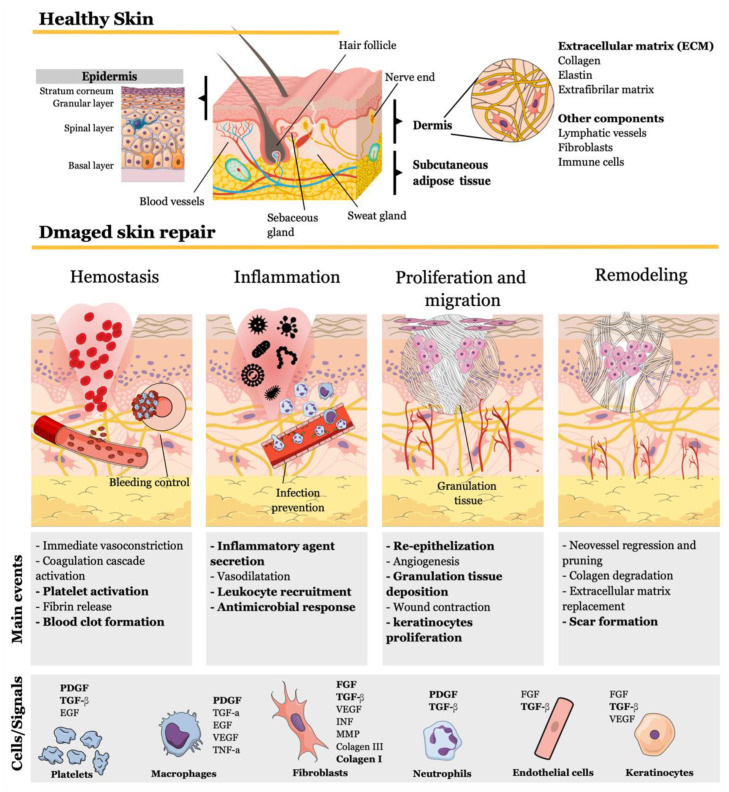
Main mechanisms involved in skin repair. Injured tissue naturally recovers through four subsequent and overlapping events orchestrated by mediator molecules secreted by different cell types to reconstitute skin structures and healthy tissue components. Chitosan interferes in several events (highlighted in bold) by stimulating signaling molecules. This figure compiled the mechanisms already described and was drawn using the infographic maker Mind the Graph available at https://mindthegraph.com.

Injured tissue can only recover with the completion of four sequential steps, which may overlap: (i) hemostasis, (ii) inflammation, (iii) migration and proliferation, and (iv) remodeling (Figure 9). This natural phenomenon immediately triggers, in addition to a pain sensation, the first mechanisms for bleeding control characterizing the hemostasis phase. Upon blood vessel rupture and dermal extracellular matrix exposure, platelets extravasate and bind to collagen, with consequent activation and degranulation, releasing multiple mediators that participate in all wound-healing steps. Local vasoconstriction is stimulated as a reflexive response triggered by endothelin secreted by damaged endothelial cells and platelet-derived growth factor (PDGF). Following platelet activation, a coagulation cascade is triggered, resulting in the conversion of fibrinogen to fibrin fibers and consequent formation of fibrin clots or thrombi to stop bleeding (Figure 9) [129,130,131,132].

After the first minutes, several proinflammatory mediators are released by injured and mast cells, including chemokines, inflammatory cytokines, hydrogen peroxide (H_2_O_2_), damage-associated molecular patterns (DAMPs), lipid mediators, vasodilator agents, vascular permeability factor, and proteases. All of these mediators promote vasodilatation, enhancing vessel permeability, as well as promoting neutrophil recruitment. These cells are attracted to the wound site and, alongside M1 macrophages, participate in the first line of defense or innate immune response against microorganisms. In response to signaling molecules secreted during the innate immune response, T lymphocytes also migrate to the wound site, representing the second line of adaptive immune response defense to ensure efficient clearance and avoid infection with the aid of antigen-presenting cells, such as B lymphocytes and dendritic cells. Under healthy physiological conditions, inflammatory reactions are terminated, and the tissue regeneration process predominates, characterizing phases iii and iv. Following inflammation, M2 macrophages, epidermal cells, platelets, and subcutaneous adipose tissue secrete vascular endothelial growth factor (VEGF), platelet-derived growth factor (PDGF), fibroblast growth factor (FGF), transforming growth factor-β (TGF-β), and angiopoietins, which stimulate endothelial cell proliferation and migration, forming new capillaries. Moreover, wound contraction is achieved by the deposition of collagen III to recover the extracellular matrix (ECM) synthesized by M2 macrophages and other fibroblasts, constituting a temporary granulation tissue. Furthermore, dermal fibroblasts respond to TNF-β secreted by epidermal stem cells differentiating to myofibroblasts, which participate in wound contraction. Tissue regeneration proceeds with re-epithelization when unipotent stem cells in hair follicles undergo proliferation alongside resident stem cells from the epidermis, followed by differentiation and migration to reconstitute epidermis cell types, including keratinocytes belonging to spinal and granular layers and cornified cells from the upper-most layer, the stratum corneum. The epidermal growth factor family regulates this stage, including EGF, TGF-α, and FGF, affecting migrating and proliferating keratinocytes. In the last phase, when the wound is already closed, regenerated tissue undergoes maturation and remodeling, lasting for months or years. New blood vessels generated during previous neovascularization are remodeled to originate mature, non-leaky, and well-perfused vessels through the regression of selected branches and a pruning process. Granulation tissue is reorganized by the replacement of collagen III with collagen I, which is stronger and lasts longer, resulting in scar formation. Collagen III is degraded through the action of matrix metalloproteinases (MMPs), which can be released by proinflammatory macrophages and fibroblasts (Figure 9) [129,130,131,132].

This natural process can be affected and delayed or impaired by local or systemic factors. Oxygenation, infection, and venous sufficiency are among the factors that can locally influence wound healing. In contrast, systemic factors, including physiological and pathological conditions, such as diabetes, uremia, cancer, and cardiovascular diseases; unhealthy behaviors, such as obesity, smoking, and alcoholism; sex hormones; nutrition status; or the use of immunosuppressing treatments, including medications such as corticosteroids and chemotherapies or radiotherapy, can also impair or delay regeneration [133]. In these cases, the use of agents able to stimulate skin repair, such as chitosan/chitin-based medical devices or topical treatment, has become a valuable strategy [129,134]. Chitosan-based material affects all wound-healing phases, leading to the acceleration of this process, culminating in the generation of chitosan-based wound dressings. The wound-healing effects of chitosan-based dressings can be modulated to speed up hemostasis or any other stage according to the structure (films, sponges, hydrogels, hydrocolloids, membranes, fibers, scaffold, and nanoparticles) and combination with different functional materials, such as gelatin, alginate, polyvinyl alcohol (PVA), carboxymethyl chitosan (CMCS), cellulose, or bioactive molecules (Table 1) [135,136].

Bleeding control can be accelerated independent of the classic coagulation system using chitosan-based material. This hemostatic effect results from electrostatic interactions between chitosan amine groups and the neuraminic acids found on erythrocyte surfaces or negatively charged molecules on platelet surfaces, promoting erythrocytes and platelet aggregation. Moreover, chitosan activates platelets through the upregulation of the membrane integrin GPIIb-IIIa glycoprotein, which is responsible for adhesion and aggregation, accelerating fibrin clot formation [129,135,137,138,139]. Chitosan foam sponge dressings reinforced with dialdehyde cellulose display superior in vitro and in vivo hemostasis capability in comparison to commercial wound dressings composed purely of chitosan, resulting in decreased blood loss and hemostasis time in rabbits and promoting erythrocyte/platelet adherence and activation, forming a robust blood clot (Table 1) [120].

Wound infection prevention can directly minimize the inflammation phase by inhibiting microorganism proliferation promoted by the antimicrobial activity of chitosan-based materials. Gram-positive and Gram-negative bacteria or fungi viability are affected by chitosan via electrostatic interactions between charged components, which include teichoic acid and lipopolysaccharides from bacteria cell walls or sialic acids that compose the carbohydrate side chains of mannoproteins in fungi cell walls with the amino groups through the molecular mechanisms represented in Figure 6 and discussed in Section 3 [14,38,129,140]. Polyvinyl alcohol/chitosan wound dressings loaded with polyhexanide inhibited in vitro *Staphylococcus aureus* and *S. epidermidis* growth. When applied to a dog’s wound, the healing process was completed, with no bacterial infection in 15 days, in contrast with conventional dressings, which did not resolve the wound, even after 21 days [118]. Mouse wounds infected with *E. coli* ATCC25922 were treated with chitosan sponge dressings loaded with Ag nanoparticles and lipopeptide iturim, resulting in faster recovery and reduced *E. coli* colony formation in the wounds compared to chitosan sponge dressing alone (Table 1) [119].

Several studies have also demonstrated the stimulation of inflammatory cells, macrophages, and fibroblasts by chitosan, boosting this stage and anticipating inflammation termination, initiating the following wound healing phases [140]. The use of chitosan scaffold dressings in the blood of patients with Von Willebrand disease (vWD), a genetic disorder that impairs hemostasis processes after tissue injury, activated platelet activity, adhesion, and aggregation, as well as the release of the critical wound-healing mediators TGF-β1 and PDGF-AB [121]. A chitosan/TiO_2_ composite membrane may be used as wound dressing, as it stimulates in vitro fibroblast proliferation, accelerating wound healing [122,136]. This property is highly influenced by the rate and degree of deacetylation, as demonstrated by Howling, Dettmar, Goddard, Hampson, Dornish and Wood [140], who observed superior proliferative rates of primary human fibroblasts upon stimulation with a highly deacetylated (86–89%) chitosan solution. Behera, Das, Kumar, Bissoyi and Singh [122] also demonstrated that a chitosan/TiO_2_ composite membrane stimulated the expression of cytokines and essential factor genes that promote faster wound healing, such as *FGF*, *TGF-β,* collagen type 1 (*COLI*), delta-like homolog 1 (*DLK1*), and proliferating cell nuclear antigen (*PCNA*) in the mouse fibroblast cell line L929. Moreover, the chitosan/TiO_2_ composite membrane also inhibited *Staphylococcus aureus* growth, one of the most common causes of wound infection (Table 1).

HaCaT keratinocytes and primary cultured keratinocytes also seem to be stimulated by chitosan-based material, but their dependence on deacetylation degree is still controversial and requires further studies [141]. A wound dressing composed of chitosan/PVA plasticized with glycerol promoted a faster healing of second-degree burn wounds in rabbits and effective inhibition of *Pseudomonas aeruginosa*, *Escherichia coli*, and *Bacillus subtillis* growth, as well as stimulation of epidermal growth and acceleration of connective tissue formation (Table 1) [123].

Chitosan wound healing potential has been confirmed in various clinical studies, which justifies its commercial utilization as wound dressing and reinforces in vitro findings. Human skin graft donor patient wounds treated with chitosan mesh membrane wound dressings exhibited improved platelet adhesion, hemostasis, granulation tissue arrangement, and faster re-epithelialization when compared to conventional dressing after two months of follow up [124]. Similarly, the efficiency of chitosan sheet dressings applied to human wounds compared to commercial dressings, alginate dressings, and silicone gauze induced complete healing in 11 days and increased dermal nerve extracellular matrix glycosaminoglycan enrichment, as well as capillaries, at the 6-month follow-up [125]. Heparin immobilization in chitosan membranes used as wound dressings in skin graft donor patients also promoted wound repair acceleration by promoting faster re-epithelialization 15 days post-treatment (Table 1) [126].

Several ongoing clinical trials worldwide aim to apply chitosan-based material alone or in combination with other agents to treat and/or prevent several diseases or physiopathological conditions, such as pressure ulcers, atopic dermatitis, knee osteoarthritis, SARS-CoV virus infection, hemostasis, bacteriostasis in cesarian sections, and lesion sterilization and tissue repair for dental treatment, as well as post-surgical peri-implant inflammation and hemostasis with consequent radial artery occlusion following transradial procedures, chondral lesions, and burn wound infection [142]. The interest in chitosan-based materials, especially patent-protected chitosan derivatives for wound-healing purposes, has increased considerably in the last ten years, resulting in 2543 new patents registered in 2020, compared to 1350 in 2010 [143].

The large number of clinical trials reinforces the plasticity of chitosan-based materials in maintaining cellular structures and tissues and protecting them (mainly skin) against external and severe injuries.

## 7. Conclusions

The use of natural bioactive compounds has increased in response to several restraints concerning environmental preservation, guaranteeing the safety of personal care products, cosmetics, cosmeceuticals, and their ingredients. However, equally importantly, these products must fulfill consumer expectations and feelings in a very competitive market with increasing pressure and growing awareness regarding the effect of the use of fossil-derived ingredients or those obtained from unsustainable raw materials. Personal care and cosmetic companies have become increasingly concerned about the ingredients used in the formulation of their products and interested in developing products from sustainable and ecological raw materials to create long-term value, preparing companies to compete in the global economy. The same is noted for cosmeceuticals, a novel class of very technologically sophisticated products categorized between cosmetics and pharmaceuticals containing bioactive compounds intended to maintain and enhance health and skin beauty. Furthermore, skin care ingredients should be devoid of genotoxic, carcinogenic, or reproductive risks, making them safe and effective. Chitosan is metabolized by humans and hydrolyzed by environmental enzymes into a linear polysaccharide that can be used as a carbohydrate source by living organisms, promoting nutrition and energy and acting as a signaling molecule.

Chitosans are unique polymeric compounds obtained from seafood waste with broad applicability. Due to their physicochemical features, variable molecular weights, and degree of deacetylation, chitosan can assume several physical forms, such as films, sponges, hydrogels, fibers, and nanoparticles. Nanocapsules and nanofilms can self-assemble following ecofriendly treatments, such as sonication, without generating secondary products. The edible nature of chitosan also allows for its inclusion as an excipient in cosmeceuticals or pharmaceuticals designed for oral administrations to protect active principles during gastrointestinal tract digestion, intestinal absorption, and when in the bloodstream environment, improving the solubility of hydrophobic compounds and controlling their release.

## 8. Future Perspectives

Despite the considerable potential of employing chitosan in its nanocounterpart form for systemic protein, as well as peptide or gene delivery, the need to develop preclinical and clinical trials has not accompanied technological development. On the other hand, the prospects for the non-parenteral application of nanochitosan and its composites in cosmetics, cosmeceuticals, and pharmaceuticals to protect, repair, and regenerate skin have developed faster, as, among novel composites, most are obtained from non-toxic compounds or natural biopolymers, and all of them are obtained from environmentally sustainable matrices. The biological effects of chitosan or nanochitosan, either intrinsic or acquired from binding or association with different composites, can affect the dermis and epidermis, avoiding blotting, inflammatory responses, and infection, re-establishing oxidative balance and allowing the skin to regenerate while also reducing scars.

## Data Availability

Data supporting reported results can be found in the manuscript.
